# Circulating miR-21 and miR-29a as Markers of Disease Severity and Etiology in Cholestatic Pediatric Liver Disease

**DOI:** 10.3390/jcm5030028

**Published:** 2016-02-25

**Authors:** Imeke Goldschmidt, Thomas Thum, Ulrich Baumann

**Affiliations:** 1 Department of Paediatric Hepatology and Gastroenterology, Hannover Medical School, Carl-Neuberg-Strasse 1, Hannover 30625, Germany; baumann.u@mh-hannover.de; 2 Institute for Molecular and Translational Therapeutic Strategies (IMTTS), Hannover Medical School, Carl-Neuberg-Strasse 1, Hannover 30625, Germany; thum.thomas@mh-hannover.de; 3 Integrated Research and Treatment Centre Transplantation (IFB-Tx), Hannover Medical School, Hannover 30625 Germany

**Keywords:** biliary atresia, hepatic fibrosis, microRNA, paediatrics

## Abstract

Circulating microRNAs have been investigated as markers of disease severity in a variety of conditions. We examined whether circulating miR-21 and miR-29a could serve as markers of hepatic fibrosis and disease etiology in children with various liver diseases. Circulating miR-21 and miR-29a were determined in 58 children (21 female, age 0.1–17.8 (median 9.8) years)) with chronic liver disease and compared to histological grading of hepatic fibrosis. 22 healthy children served as controls for circulating miRNAs. Levels of circulating miR-21 appeared to be age-dependent in healthy children. Children with biliary atresia had significantly higher levels of miR-21 compared both to healthy controls and to age-matched children with other cholestatic liver disease. Circulating miR-29a levels in biliary atresia children did not differ from healthy controls, but tended to be higher than in age-matched children with other cholestatic liver disease. Neither miR-21 nor miR-29a correlated well with hepatic fibrosis. Circulating miR-21 and miR-29a levels can potentially serve as non-invasive diagnostic markers to differentiate biliary atresia from other cholestatic disease in infancy. They do not appear suitable as non-invasive markers for the degree of hepatic fibrosis in an unselected cohort of children with various liver diseases. The discriminating effect regarding neonatal cholestasis should be followed up in a prospective longitudinal study.

## 1. Introduction

microRNAs are small (18–23 nucleotides), non-coding RNA molecules involved in regulation and stabilization of mRNA translation [1] that have been shown to play a pathogenetic role in several disease entities [2,3,4]. Circulating microRNAs appear to be relatively stable in the peripheral blood and in urine [5], and have been examined as disease-specific biomarkers in conditions as diverse as Crohn’s disease [6] and hepatocellular carcinoma (HCC) [4].

In adult hepatology, microRNAs have been extensively investigated in the context of non-alcoholic fatty liver disease and HCC [4]. Knowledge on microRNA differential regulation in pediatric liver disease is still scarce. MicroRNAs have been investigated as potential markers of acetaminophen-toxicity in children [7]. miR-122, miR-25, and miR-21 were recently described as potential markers of cystic-fibrosis-related liver disease in children [8]. MicroRNA panels to facilitate non-invasive diagnosis of hepatic malignancy in in children are currently being evaluated [9]. Two recent publications by Hand [10] and Zahm *et al.* [11]*,* demonstrated differential regulation of miR-21 and -29a during the development of biliary atresia in a murine model [10], and identified miR-29a as a potential disease-specific marker for biliary atresia in infants [11]. miR-29a has previously been described as a diagnostic marker in both experimental hepatic fibrosis in mice, and in advanced hepatic fibrosis in adults [3]. It has also been shown to have a protective role against liver fibrosis in a mouse model of obstructive jaundice [12]. miR-21 has been linked to the development of atrial fibrosis in the heart [2,13].

In children, a non-invasive diagnostic alternative for the diagnosis of liver disease and hepatic fibrosis appears particularly attractive, since liver biopsy is invasive, and is fraught with sampling error due to small sample sizes. The distinction of biliary atresia (BA) from other causes of neonatal cholestasis presents a particularly challenging clinical problem. Given the new diagnostic potential of circulating microRNAs, the aim of our study was to explore the potential diagnostic role of circulating miR-21 and miR-29a for the diagnosis of hepatic fibrosis in pediatric liver disease, with a focus on differentiating pediatric cholestatic liver diseases.

## 2. Materials and Methods

### 2.1. Subjects

58 children (21 female, age 0.1–17.8 (median 9.8) years) with chronic liver disease (biliary atresia (BA) *n =* 9, autoimmune hepatitis (AIH) *n =* 7, non-alcoholic fatty liver disease (NAFLD) *n =* 6, primary-sclerosing cholangitis (PSC) *n =* 6, progressive familial intrahepatic cholestasis (PFIC) *n =* 5, after liver transplantation (OLT) *n =* 10, and miscellaneous or unknown etiology *n =* 15) underwent blood sampling for circulating microRNAs at the occasion of a clinically-indicated liver biopsy. 22 healthy children (nine female, age 0.16–16.8 (Median 6.3) years) who underwent blood sampling at the occasion of minor surgical procedures served as controls. All parents of patients and controls gave informed consent for study participation. The study was conducted in compliance with the Declaration of Helsinki, and the study protocol was approved by the local ethics board (Ref. N° 5177)

### 2.2. Liver Histology

Liver fibrosis was graded according to ISHAK on hematoxylin-eosin stained biopsy samples.

### 2.3. Circulating microRNAs

Serum samples for microRNA analysis were stored at −20 °C until analysis. Serum concentrations of miR-21 and miR-29a were determined by reverse transcription PCR. In brief, RNA was isolated from serum with Master Pure RNA Purification Kit (Epicentre, Madison, WI, USA) and reverse transcribed into cDNA. Defined quantities of *Caenorhabditis* *elegans* miR-39 were added for normalization purposes. Quantitative PCR was performed using iQ supermix (Bio-Rad, Hercules, CA, USA) and miRNA-specific TaqMan hybridization probes (Applied Biosystems, Foster City, CA, USA). Each sample was measured twice and mean cyclic threshold (*ct*) values of both measurements were used for all following transformations. Delta *ct* values (miR-21/miR-29a *vs.* cel-miR-39) were calculated for all patient and control samples. Measurements were performed in two separate runs. A subset of eight sera from healthy children spanning the complete age range of patients and healthy controls alike (normalization group) was analyzed in both RT-PCR runs. To facilitate comparison between the different RT-PCR runs, delta-delta *ct* values were calculated for each patient and control sample using the delta *ct* value of the respective sample, and the mean delta *ct* value of the normalization group.

### 2.4. Statistics

Quantitative variables are given as mean or median (range) as appropriate. Comparison between quantitative variables is made using student’s *t*-test. Correlation between quantitative variables is calculated using Pearsson’s correlation coefficient; while correlation with parametric data (ISHAK score) is calculated using Kendall-Tau-b correlation. Analysis of miR-21 and miR-29a values in our control group showed that miR-21, but not miR-29a, levels displayed a significant negative correlation with age (*r* = −0.53, *p* = 0.002, and *r* = −0.29, *p* = 0.13 respectively). Therefore, for all miR-21 calculations, an age-matched subset of healthy control values was used for comparison with patient samples. Comparison of variables between groups was made using student’s *t*-test or Mann–Whitney *U*-test, as appropriate.

## 3. Results

Our first question was whether circulating miR-21 and miR-29a could serve as a marker of hepatic fibrosis, independent of disease etiology. Correlation of miR-21 and miR-29a with hepatic fibrosis as measured by ISHAK score was 0.029 (n.s.) and −0.09 (n.s.), respectively, indicating no diagnostic use in an unselected patient sample. Analysis in the various diagnostic subgroups (BA, AIH, PSC, NAFLD/NASH, OLT) did not reveal any disease-specific differences (Table S1).

**Figure 1:**
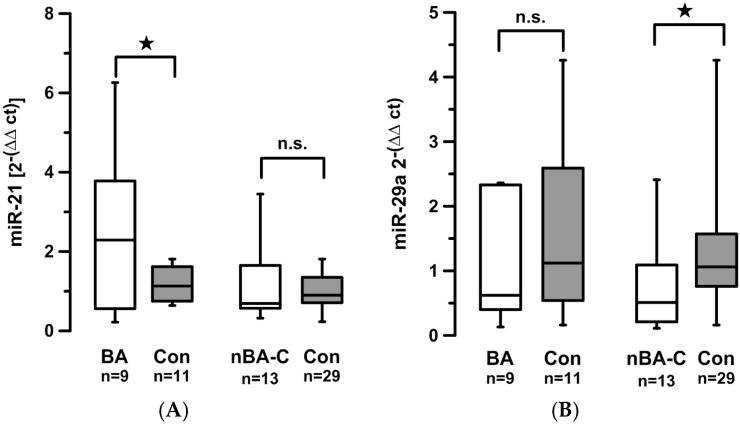
Circulating levels of miR-21 (**A**) and miR-29a (**B**) are compared between children with biliary atresia (BA), children with cholestatic disease other than biliary atresia (non-BA), and healthy controls (Con). ★ indicates *p* < 0.05. n.s.: not statistically significant.

Our second question was whether levels of circulating miRNAs might be specific for disease entities. In patients with BA, miR-21 levels were significantly higher than in aged-matched healthy controls (2.4 *vs.* 1.2, *p* = 0.045) (Figure 1A). Levels of circulating miR-29a did not differ significantly between BA patients and controls (0.98 *vs.* 1.55, *p* = 0.24) (Figure 1B).

In order to test whether this increase in circulating miR-21 is specific to BA or whether it might rather be an effect of cholestasis *per se*, we compared miRNA-levels between children with BA and children with other cholestatic diseases (bilirubin levels above 50 µmol/L; for a full list of patients see Table S2). In non-BA cholestatic children (nBA-C), miR-21 levels did not differ significantly from controls. However, miR-29a levels were significantly lower compared to controls (0.72 *vs.* 1.26, *p =* 0.04) (Figure 1).

**Figure 2:**
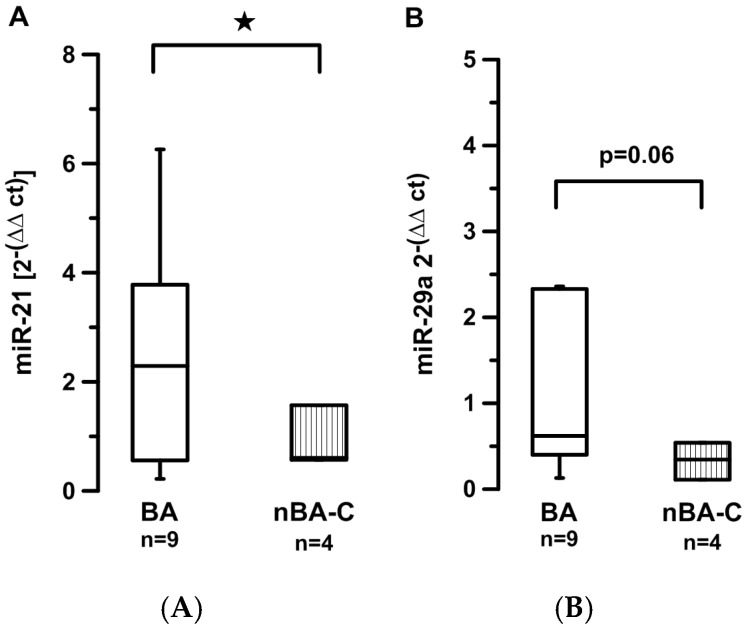
Circulating levels of miR-21 (**A**) and miR-29a (**B**) are compared between children with biliary atresia (BA) and age-matched children with cholestatic disease other than biliary atresia (nBA-C). ★ indicates *p* < 0.05.

Comparing BA *vs.* nBA-C children directly was rendered difficult by the difference in age between the two groups (in our sample BA median age 0.3 years (range 0.1–0.78) (*n =* 9), nBA-C median age 12 years (range 0.16–17.8) (*n =* 13)). Age-adaptation of the nBA-C group left only four children in this group. Still, miR-21 levels were significantly higher in the BA group (2.4 *vs.* 0.8, *p =* 0.48) (Figure 2A), and miR-29a levels tended to be higher in the BA group (0.98 *vs.* 0.33, *p =* 0.06) (Figure 2B). The difference could not be explained by differences in the degree of hepatic fibrosis (ISHAK BA: Median 3, range 2–5; ISHAK non-BA-C: Median 3, range 2–5.5; *p =* 0.9).

**Figure 3:**
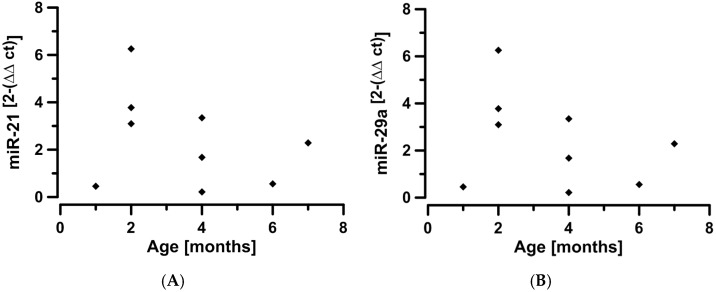
Circulating miR-21 (**A**) and miR-29a (**B**) are plotted against age in biliary atresia children. While these represent cross-sectional, not longitudinal data, there still appears to be a change in miRNA expression over time that potentially correlates with stage of disease.

Plotting miRNA-levels against age in the BA group suggest an influence of time and, possibly, stage of disease (Figure 3).

No difference between patient and control samples was found for circulating miR-21 in AIH, NAFLD/NASH, and PSC. There was a trend for lower levels of miR-29a in patients with AIH and PSC, but this failed to reach clear statistical significance (Table 1). Between these disease entities, no significant differences were found in circulating miR-21 and miR-29a levels (Table S3).

## 4. Discussion

Circulating levels of microRNAs have recently been demonstrated to be of diagnostic value in liver diseases such as NAFLD/NASH and HCC [4]. We were interested to see whether circulating miRNAs could also be of diagnostic value in the context of pediatric liver disease—more specifically in the assessment of hepatic fibrosis and in the differentiation of disease etiologies in children with liver disease. miR-21 and miR-29a were chosen as both have previously been described to be linked to organ fibrosis [2,3,12,14]. In children, miR29a has been described as a potential marker for biliary atresia [10,11].

In contrast to adult and experimental data [3,12], in our cohort neither miR-21 nor miR-29a correlated with the histological degree of fibrosis as determined by the ISHAK scoring system. Roderburg *et al.* described down-regulation of miR-29a in hepatic fibrosis in adults to be dependent on disease etiology, with lowest levels seen in alcoholic and biliary cirrhosis. An influence of disease etiology in our rather mixed cohort is conceivable and might explain the lack of correlation observed. Analysis according to disease subgroup did not yield any different results; however, these individual groups might have been too small to show a significant influence. For miR-21, the dependence on age which we detected for circulating miR-21 in the healthy control cohort might represent an additional confounder. Our patient cohort spans the entire pediatric age range, but is too small to allow for separate analysis according to age groups to verify this assumption.

The most striking result of our work was the elevation of miR-21 in children with biliary atresia compared to age-matched controls and to age-matched children with cholestatic disease other than BA. This is in contrast to the results of Hand *et al.* [10]*,* who described elevation of miR-21 in the course of the development of biliary atresia in the murine mouse model [10], but could not demonstrate elevation of circulating miR-21 in a large sample of children with biliary atresia [11]. In contrast, results for circulating miR-29a with a trend to be higher in children with BA as opposed to non-BA cholestatic children) were similar between our study and the results of the group of Hand and Zahm *et al.* [11]. Based on the work of Zahm *et al.*, the measurement of miR-200b/429 as an additional marker might provide useful additional information. Since these results were published after completion of our study and there is no material left of the samples used in our work, this question will need to be addressed in a later study.

The main source of potential bias and, hence, the potential basis for conflicting results, could be the timing of the sampling. The difficulty results from taking a cross-sectional viewpoint in what is really a longitudinal, time-dependent process. Hand *et al.* [10] demonstrated a definite time-dependency of miR-21 expression in BA mice. Looking at our age *vs.* miRNA-level plot, there also appears to be a reduction with time. The majority of our BA patients was 2–4 months old (1 month *n =* 1, 2 months *n =* 3, 4 months *n =* 3. 6 and 7 months *n =* 1, respectively). None had received a Kasai operation at the time of sampling. We do not know the age distribution of the patient sample used for the study of Zahm *et al.* [11], but it appears conceivable that time and disease stage have a major influence on results. A prospective study in human infants presenting with neonatal cholestasis, incorporating serial measurements of circulating miRNA and linking the findings to both histological and clinical staging of disease severity, should be able to overcome this limitation. Our data suggest that not only circulating miR-29a, but also circulating miR-21 might be a useful diagnostic marker for biliary atresia. This could potentially provide a breakthrough in the clinical challenge presented by having to make a definite diagnosis in an infant with neonatal cholestasis under the pressure of ascertaining the indication for a Kasai operation.

## 5. Conclusion

Circulating miR-21 levels are higher in infants with biliary atresia compared to healthy children and age-matched patients with other cholestatic disease. MiR-29a levels are reduced in non-biliary atresia children compared to healthy controls. Both miR-21 and miR-29a do not appear suitable as non-invasive markers for the degree of hepatic fibrosis in an unselected cohort of children with various liver diseases.

A prospective longitudinal study in infants presenting with neonatal cholestasis should be able to differentiate the influences of disease etiology and disease stage on circulating miRNA levels, with the potential opportunity to deliver a non-invasive diagnostic tool for BA in the future.

## Figures and Tables

**Table 1:** Circulating miR-21 and miR-29a levels in various etiological groups.

	miR-21 [2^(−ΔΔ*ct*)^] Median (Range)	Disease *vs.* Control *t*-test/MWU	miR29a [2^(−ΔΔ*ct*)^] Median (Range)	Disease *vs.* Control *t*-test/MWU
Controls for AIH (*n =* 17)	0.78 (0.23–1.53)		0.92 (0.20–2.37)	
Controls for AFLD/NASH & PSC (*n =* 11)	0.74 (0.23–1.27)		0.86 (0.20–2.37)	
AIH (*n =* 7)	0.72 (0.50–1.73)	*p =* 0.69/*p =* 0.85	0.67 (0.21–1.26)	*p =* 0.088/*p =* 0.15
NAFLD/NASH (*n =* 6)	0.82 (0.36–1.78)	*p =* 0.21/*p =* 0.15	0.85 (0.31–1.49)	*p =* 0.76/*p =* 0.88
PSC (*n =* 6)	0.44 (0.32–1.24)	*p =* 0.52/*p =* 0.18	0.37 (0.13–1.0	*p =* 0.05/*p =* 0.06

## Supplementary Files

Supplementary File 1
