# Supplementary material for: Circulating miR-21 and miR-29a as Markers of Disease Severity and Etiology in Cholestatic Pediatric Liver Disease
Source: J Clin Med. 2016 Feb 25;5(3):28. doi: 10.3390/jcm5030028 (PMC4810099; doi:10.3390/jcm5030028)
Supplement: Supplementary File 1 [file jcm-05-00028-s001.docx]

**Supplementary Materials: Circulating miR-21 and miR-29a as Markers of Disease Severity and Etiology in Cholestatic Pediatric Liver Disease**

Imeke Goldschmidt, Thomas Thum and Ulrich Baumann

**Table S1.** Correlation of circulating miR-21 and miR-29a with ISHAK score according to disease etiology.

| **Diagnosis** | ***n*** | **miR-21** | ***p*** | **miR-29a** | ***p*** | **ISHAK Range** |
| --- | --- | --- | --- | --- | --- | --- |
|  |  | **Kendall-Tau-b** |  | **Kendall-Tau-b** |  |  |
| Biliary atresia | 9 | −0.3 | 0.91 | −0.09 | 0.75 | 2–5 (Median 3) |
| Cholestasis other than BA | 13 | −0.32 | 0.14 | −0.19 | 0.39 | 0–6 (Median 3) |
| PSC | 6 | −0.28 | 0.44 | −0.28 | 0.44 | 0–6 (Median 2) |
| NAFLD/NASH | 6 | 0.29 | 0.42 | 0.15 | 0.69 | 0–5 (Median 1) |
| AIH | 7 | 0 | 1 | −0.41 | 0.21 | 1–5 (Median3) |
| OLT | 10 | −0.44 | 0.1 | −0.18 | 0.5 | 0–1 (Median 0) |

**Table S2.** List of diagnoses in non-BA cholestatic children.

| **Diagnosis** | **Age** | **Bilirubin** |
| --- | --- | --- |
|  | [months/years] | [µmol/L] |
| Unknown | 6 months | 71 |
| Alagille | 2 months | 66 |
| Neonatal cholestasis, not BA | 3 months | 80 |
| Neonatal cholestasis, not BA | 4 months | 207 |
| PFIC | 1.4 years | 393 |
| OLT | 2.6 years | 104 |
| Unknown | 16.2 years | 134 |
| BRIC | 15.4 years | 354 |
| Unknown | 16.1 years | 221 |
| AIH | 10.9 years | 68 |
| PSC | 13.1 years | 107 |
| PSC | 16.4 years | 50 |
| PFIC | 17.8 years | 286 |
| Bilirubin levels in BA children |  |  |
| Bilirubin [µmol/L] | 202 (98–549) | Mean (range) |

**Table S3.** Comparison of circulating levels of miR-21 and miR-29a between different disease etiologies.

| ***p* Values** | **miR-21** | **miR29a** |
| --- | --- | --- |
| (Mann–Whitney-*U*-test) |  |  |
| AIH *vs.* PSC | 0.07 | 0.29 |
| AIH *vs.* NAFLD/NASH | 0.73 | 0.63 |
| NAFLD/NASH *vs.* PSC | 0.09 | 0.09 |
